# Effects of Transcranial Direct Current Stimulation (t-DCS) of the Cerebellum on Pain Perception and Endogenous Pain Modulation: a Randomized, Monocentric, Double-Blind, Sham-Controlled Crossover Study

**DOI:** 10.1007/s12311-022-01498-x

**Published:** 2022-12-08

**Authors:** Regina Stacheneder, Laura Alt, Andreas Straube, Ruth Ruscheweyh

**Affiliations:** 1 Department of Neurology, University Hospital Großhadern, Ludwig-Maximilians-University Munich, Marchioni-Str. 15, 81377 Munich, Germany; 2 Department of Neurology, University Hospital Mannheim, 68167 Mannheim, Germany; 3 Department of Neurology, Ulm University Hospital, 89081 Ulm, Germany; 4 Graduate School of Systemic Neurosciences, Ludwig-Maximilians-University Munich, Planegg-Martinsried, Germany; 5 Research Training Group 2175, Ludwig-Maximilians-University Munich, Planegg-Martinsried, Germany

**Keywords:** Cerebellum, Neuromodulation, t-DCS, Pain, Offset analgesia, Non-invasive brain stimulation

## Abstract

Accumulating evidence demonstrates a role of the cerebellum in nociception. Some studies suggest that this is mediated via endogenous pain modulation. Here, we used t-DCS to test the effects of modulation of cerebellar function on nociception and endogenous pain modulation. Anodal, cathodal, and sham cerebellar t-DCS were investigated in a cross-over design in 21 healthy subjects. The nociceptive flexor (RIII) reflex, conditioning pain modulation (CPM), and offset analgesia (OA) paradigms were used to assess endogenous pain modulation. Somatosensory evoked potentials (SEPs) and pain ratings were used to assess supraspinal nociception and pain perception, respectively. No significant t-DCS effects were detected when including all t-DCS types and time points (baseline, 0, 30, 60 min post t-DCS) in the analysis. Exploratory analysis revealed an increased RIII reflex size immediately after cathodal t-DCS (compared to sham, *P* = 0.046, *η*^2^_*p*_ = 0.184), in parallel with a trend for a decrease in electrical pain thresholds (*P* = 0.094, *η*^2^_*p*_ = 0.134), and increased N120 SEP amplitudes 30 min after cathodal compared to anodal t-DCS (*P* = 0.007, *η*^2^_*p*_ = 0.374). OA was increased after anodal compared to sham stimulation (*P* = 0.023, *η*^2^_*p*_ = 0.232). Exploratory results suggested that cathodal (inhibitory) cerebellar t-DCS increased pain perception and reduced endogenous pain inhibition while anodal (excitatory) t-DCS increased endogenous pain inhibition. Results are principally compatible with activation of endogenous pain inhibition by cerebellar excitation. However, maybe due to limited t-DCS skull penetration, effects were small and unlikely to be clinically significant.

**Supplementary Information:**

The online version contains supplementary material available at 10.1007/s12311-022-01498-x.

## Introduction

The cerebellum is primarily known for its function in motor coordination, but its role in pain processing has been the focus of recent studies [1, 2].

Animal studies have shown that the cerebellum receives nociceptive input via nociceptive Aδ- and C-fibers [3, 4]. Functional MRI demonstrates cerebellar activity in response to experimental and clinical pain, especially in ventral regions [5]. Clinically, patients after cerebellar infarction exhibit increased pain perception [2].

Therefore, modulation of cerebellar activity has been tried with the goal to influence pain processing. Transcranial direct current stimulation (t-DCS) offers a safe and noninvasive way to modulate superficial cortex areas, where anodal and cathodal stimulation usually lead to neuronal excitation and inhibition, respectively [6, 7]. t-DCS over the primary motor or somatosensory cortex is able to modulate pain [8–10]. Some influence of cerebellar t-DCS on pain perception has also been reported [11–13]. Although the direction of t-DCS effect is difficult to predict because of the complex cerebellar anatomy, anodal stimulation seems to lead to pain reduction while cathodal stimulation does the opposite [11–13]. The proposed mechanism is that anodal stimulation strengthens the so-called cerebellar brain inhibition by excitation of inhibitory Purkinje cells relayed through cerebellar nuclei and thalamus to different cortical areas [11, 12, 14]. In addition, animal studies have suggested a role of pain modulatory pathways descending towards the spinal dorsal horn “descending pain modulation” [15, 16], maybe via connections between the cerebellum and the dorsolateral prefrontal cortex [17, 18] and/or the periaqueductal gray (PAG) [19, 20], which are key structures of the descending pain modulatory pathways [21, 22]. Indeed, altered endogenous pain modulation could be demonstrated also in patients after cerebellar infarction [2].

To investigate if modulation of cerebellar function affects endogenous (including descending) pain modulation (as quantified by RIII reflex, offset analgesia, CPM effect) in addition to pain perception (pain ratings) and supraspinal nociception (as quantified by SEPs), we used anodal, cathodal, and sham cerebellar t-DCS in healthy subjects. Our hypothesis, based on the considerations above, was that anodal t-DCS would reduce pain perception and increase endogenous pain inhibitory mechanisms, while cathodal t-DCS would have opposite effects [11, 15].

## Methods

### Participants

The study was conducted in accordance with the Declaration of Helsinki and approved by the ethics committee at LMU Munich (18–328). All subjects gave written informed consent. Healthy participants were recruited through university campus and social media advertisements between August 2018 and February 2019. Inclusion criteria were as follows: (1) adult with sufficient knowledge of German; (2) no psychiatric, neurological, or internal diseases, substance abuse, or chronic pain; (3) no contraindications for cranial t-DCS (active implants (e.g., pacemakers), cranial bone gaps, bone plates or screws, or local skin abnormalities); (4) not pregnant or breast-feeding; (5) no acute/chronic pain or pain medication within 2 days prior to participation.

Expected effect sizes were unknown, especially for the effect of t-DCS on endogenous pain modulation. We therefore based the sample size on our earlier work showing the influence of different interventions on spinal nociception as quantified by the RIII reflex (*n* = 15) [23] and on previous studies demonstrating the effect of cerebellar t-DCS on pain (*n* = 14–16) [11, 12, 24] and added a 30% safety margin, resulting in a target sample size of 21 participants. Twenty-seven t-DCS-naïve subjects attended the preparatory session. Six were excluded because the RIII reflex was < 200 µV*ms at tolerable stimulation intensities, leaving 21 subjects for analysis. Numbers were lower for SEPs (*n* = 18, due to muscle artifacts) and for RIII thresholds (*n* = 19, due to technical difficulties).

### Study Design

The study was randomized, double-blinded, sham-controlled and cross-over and consisted of one preparatory and three experimental sessions. In the preparatory session, subjects were acquainted with RIII recording, pain intensity rating (NRS), and the CPM test. The experimental sessions were identical apart from type of t-DCS (anodal, cathodal, or sham in randomized order) and at least 7 days apart.

**Fig. 1:**
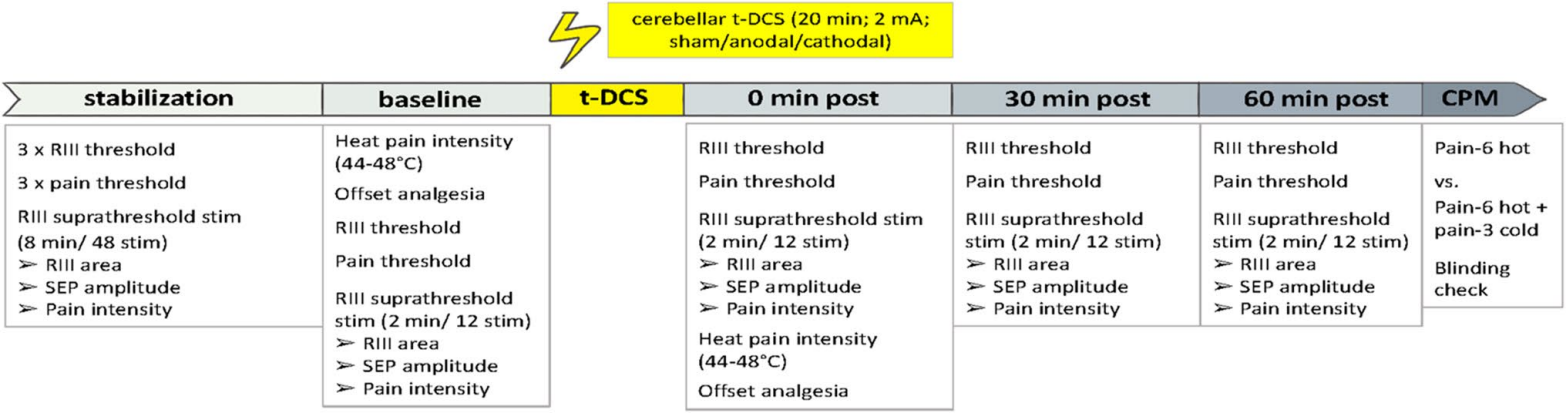
Outline of experimental procedures. The three experimental sessions were identical apart from type of t-DCS (anodal, cathodal, or sham in randomized order). The stabilization period was used to establish a steady RIII response, subsequent measurements (baseline, 0/30/60 min post and CPM), during which the different methods described above were used, results recorded and evaluated after each session

We assessed t-DCS effects on a wide range of pain and pain modulation measures at 0, 30, and 60 min after stimulation (“post”), compared to baseline [7]. Please see Fig. 1 for a list of measurements performed at every time point. Heat pain ratings and offset analgesia were obtained only at baseline and 0 min post to avoid habituation to heat stimuli [25] and CPM was only measured once at the end of the session to avoid carry-over effects of the conditioning stimulus. Finally, participants rated if they believed to have received true or sham t-DCS (blinding check).

### t-DCS

We used the neuroConn DC-Stimulator Plus by NeuroCare (Munich, Germany) with 5 × 7 cm electrodes (rubber electrodes placed within sponges soaked with isotonic salt solution) centered at the midline, 1–2 cm below the inion (active) and on the lateral upper arm (reference). t-DCS was applied for 20 min at 2 mA [7]. Stimulation sites and parameters were chosen according to the previous study with the largest effects of cerebellar t-DCS on pain [11]. Investigator (RS) blinding was achieved using the device’s study mode that selects anodal, cathodal, or sham stimulation based on 5-digit codes provided on a randomization list (generated by RR). t-DCS starts with a ramp-up phase to reach the target current (15 s). To maintain blinding during sham stimulation, sham stimulation consisted of the same ramp-up phase (to evoke the typical initial itching and tingling sensations), immediately followed by a ramp-down phase (15 s), as described in the literature [26].

### RIII Reflexes, SEPs, and Pain Ratings

RIII reflex and SEP recordings were performed as described in detail previously [27]. Briefly, participants sat in a reclining chair with the knee flexed at ~ 150°. A Keypoint Portable EMG System (Natus, Planegg, Germany) was used for stimulation of the sural nerve at the lateral malleolus (bar electrode) and recording from the short head of the biceps femoris (RIII reflex, Ag/AgCl surface electrodes) and from the vertex (Cz) with reference to the forehead (Fpz, SEPs, standard EEG electrodes). Each stimulus consisted of 5 impulses (1 ms, 200 Hz). Signals were amplified (up to 10,000 times), band-pass filtered (20 to 1000 Hz for RIII; 0.5 to 500 Hz for SEP), and saved for offline analysis.

Each 2-min cycle of suprathreshold RIII reflex recording (Fig. 1) consisted of 12 stimuli at 8–12-s intervals given at ~ 180% RIII threshold (see below). RIII reflex signals were rectified; the area under the curve (90–150 ms after stimulation) was baseline-corrected (65–5 ms before stimulation) and averaged over the 12 stimuli.

SEP traces were visually examined and discarded if the amplitude exceeded 100 µV or artifacts were present. The remaining 10 to 12 traces were averaged for each 2-min cycle, and 4 average amplitudes were extracted using the following analysis windows: 35–50 ms after stimulation for the P45 peak, 70–100 ms for the N100, 100–150 ms for the N120, and 280–350 ms for the P260 peak, according to our previous procedure [27].

The average pain intensity of the last 5 electrical sural nerve stimuli was rated at the end of each 2-min cycle on the NRS (0–10, 0 = no pain, 10 = strongest imaginable pain).

### RIII Reflex Thresholds and Pain Thresholds

Thresholds were determined using an established protocol [28]. An RIII reflex was detected if the mean EMG response between 90 to 150 ms after stimulation exceeded 1.5 times the baseline standard deviation (65 to 5 ms before stimulation). An up- and down method was used at 0.5-mA steps and the average of 4 threshold values was calculated. The same protocol was used for pain thresholds, with pain detected at an NRS ≥ 1.

### Heat Pain Ratings, Offset Analgesia, and CPM Effect

For application of contact heat, the Pathway Pain & Sensory Evaluation System with a 3 × 3 cm ATS-thermode (Medoc, Israel) was used with a baseline temperature of 32 °C and temperature ramps of 8°/s. The thermode was shifted between stimulations.

To obtain heat pain intensity ratings, 5-s heat stimuli at 44, 45, 46, 47, and 48 °C were applied to the lateral upper right and left arm in random order. NRS ratings were averaged over the two arms [29].

Offset analgesia was assessed as described previously [2]. Briefly, 30-s heat stimuli (3 control, 3 offset, in randomized order) were applied to the volar forearm at a predetermined target temperature evoking a pain sensation of ~ 5 on the NRS (pain-5 hot, 45.2 ± 1.2 °C) and rated on the NRS every 5 s, starting at 4 s. Control stimuli were kept constant, while offset stimuli increased by 1 °C after 5 s and returned to target after another 5 s. Average ratings were then expressed in percent of the first (4 s) rating. “Offset minus control” percent difference scores were calculated, and offset analgesia was quantified as the difference score at 14 s [2].

Conditioned pain modulation (CPM) was measured using an established protocol [30], using a 30-s test stimulus individually pre-determined to evoke a painful sensation at NRS ~ 6 (pain-6 hot, 46.3 ± 1.2 °C) and a 60-s conditioning stimulus pre-determined to evoke a painful sensation at NRS ~ 3 (pain-3 cold, 9.2 ± 1.7 °C, cold water in a Styrofoam box up to the wrist). The test stimulus was applied twice to the left dorsal forearm, once before and once starting 30 s into the application of the conditioning stimulus at the contralateral hand. Test stimuli were rated on the NRS every 10 s, and the CPM effect was calculated as the difference between the average test pain ratings during minus before conditioning stimulus. A negative CPM effect indicates an activation of pain inhibitory mechanisms.

### Statistical Analysis

Analysis was performed with IBM SPSS Statistics version 27 (IBM®, Armonk, NY, USA). Values are mean ± standard deviation unless indicated otherwise. *P* < 0.05 was considered significant. t-DCS effects were tested using repeated-measures Analysis of variance (ANOVA) with the factors t-DCS type (anodal, cathodal, sham) and time (time points as applicable), followed by subordinate ANOVAs and post hoc tests where applicable. Only results for the effect of interest (interaction between t-DCS and time) are shown. T-DCS effects on CPM were assessed using ANOVA with the factors t-DCS type and condition (before/during conditioning stimulation). In case of violation of sphericity, Greenhouse–Geisser correction was applied. The blinding check was tested using chi-square test.

## Results

Analysis is based on 21 participants (23.0 ± 3.9 years, 76.2% females).

### RIII Reflex Areas and Corresponding Pain Ratings

**Fig. 2:**
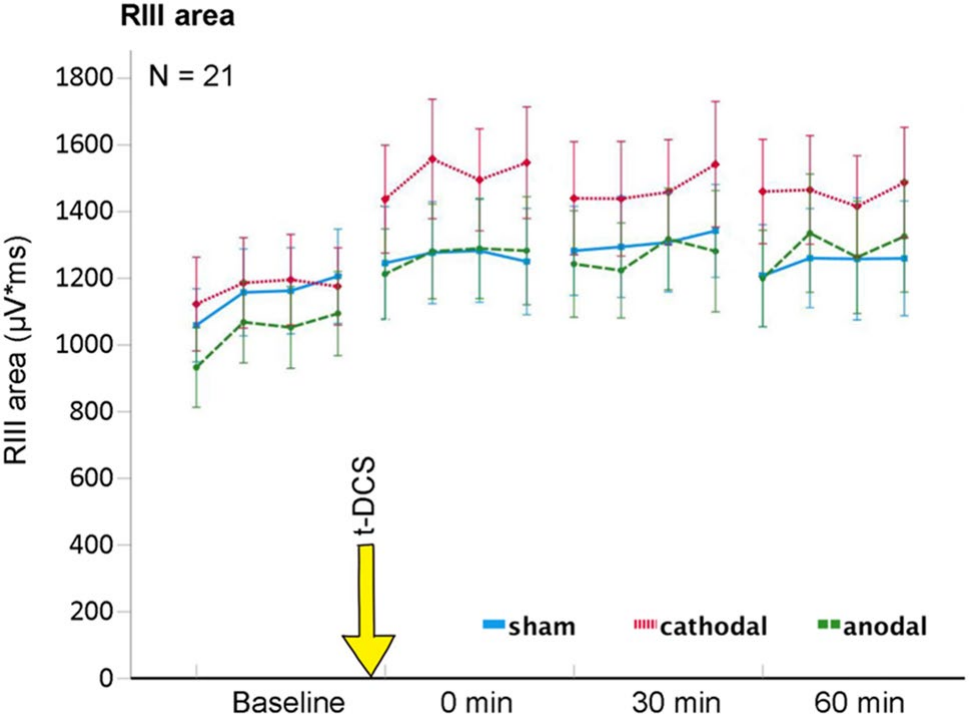
Effect of t-DCS (anodal/cathodal/sham) on RIII reflex areas. Each data point represents an average of 3 RIII reflexes and 21 subjects. Exploratory analysis revealed a significant increase in the RIII area from baseline to 0 min post after cathodal compared to sham stimulation (*P* = 0.046)

Results are shown in Fig. 2 and Table 1. When considering all 4 time points, ANOVA identified no significant interaction between time and t-DCS type (*F* [6,120] = 0.667; *P* = 0.677). However, visual inspection suggested increased RIII areas immediately after cathodal t-DCS (Fig. 2). Indeed, exploratory analysis indicated a significant increase in RIII areas after cathodal compared to sham t-DCS at 0 min post compared to baseline (*F* [1, 20] = 4.507; *P* = 0.046) that amounted to 132.7% ± 34.6% of baseline (compared to 109.7% ± 26.4% in the sham group). Pain ratings of the RIII-evoking electrical stimulus showed no significant interaction between t-DCS type and time (*F* [6,120]  = 1.168; *P* = 0.331).

**Table 1:**
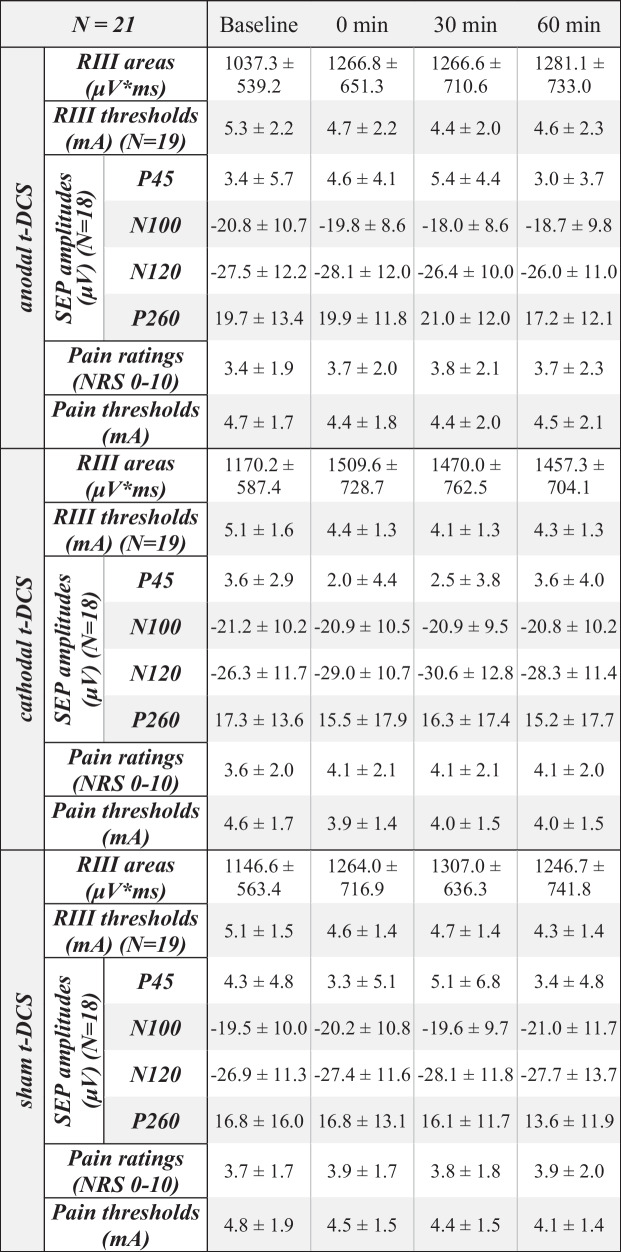
T-DCS effects on RIII reflexes, SEPs, and electrical pain ratings. Values are mean ± standard deviation

### RIII Reflex Thresholds and Pain Thresholds

Results are shown in Table 1. There was no significant interaction between time and t-DCS type on RIII thresholds (*F* [6,102] = 1.611; *P* = 0.189) or pain thresholds (*F* [6,120] = 1.440; *P* = 0.205). Visual inspection suggested a reduction of pain thresholds immediately after cathodal stimulation. Exploratory analysis revealed a trend for decreased pain thresholds (*F* [1, 20] = 3.092; *P* = 0.094) from baseline to 0 min post in cathodal (− 0.63 mA ± 0.89 mA) compared to sham (− 0.33 mA ± 0.80 mA) stimulation.

### SEP Amplitudes

Results are shown in Supp. Figure 1 and Table 1. There were no significant interactions between time and t-DCS type (P45: *F* [6,96] = 0.661, *P* = 0.681; N100: *F* [6,96] = 0.785, *P* = 0.583; N120: *F* [6,96] = 1.735, *P* = 0.121; P260: *F* [6,96] = 0.168, *P* = 0.985). Visual inspection suggested an increased N120 component 30 min after cathodal t-DCS. Indeed, exploratory analysis showed a significantly increased N120 amplitude after cathodal (4.3 µV ± 7.5 µV) compared to anodal stimulation (− 1.1 µV ± 4.5 µV) from baseline to 30 min post (*F* [1, 16] = 9.557; *P* = 0.007).

### Heat Pain Intensity Ratings

There was no significant interaction of time and t-DCS type on heat pain intensity ratings (*F* [8,160] = 0.437; *P* = 0.766, Supp. Table 1).

### Offset Analgesia

**Fig. 3:**
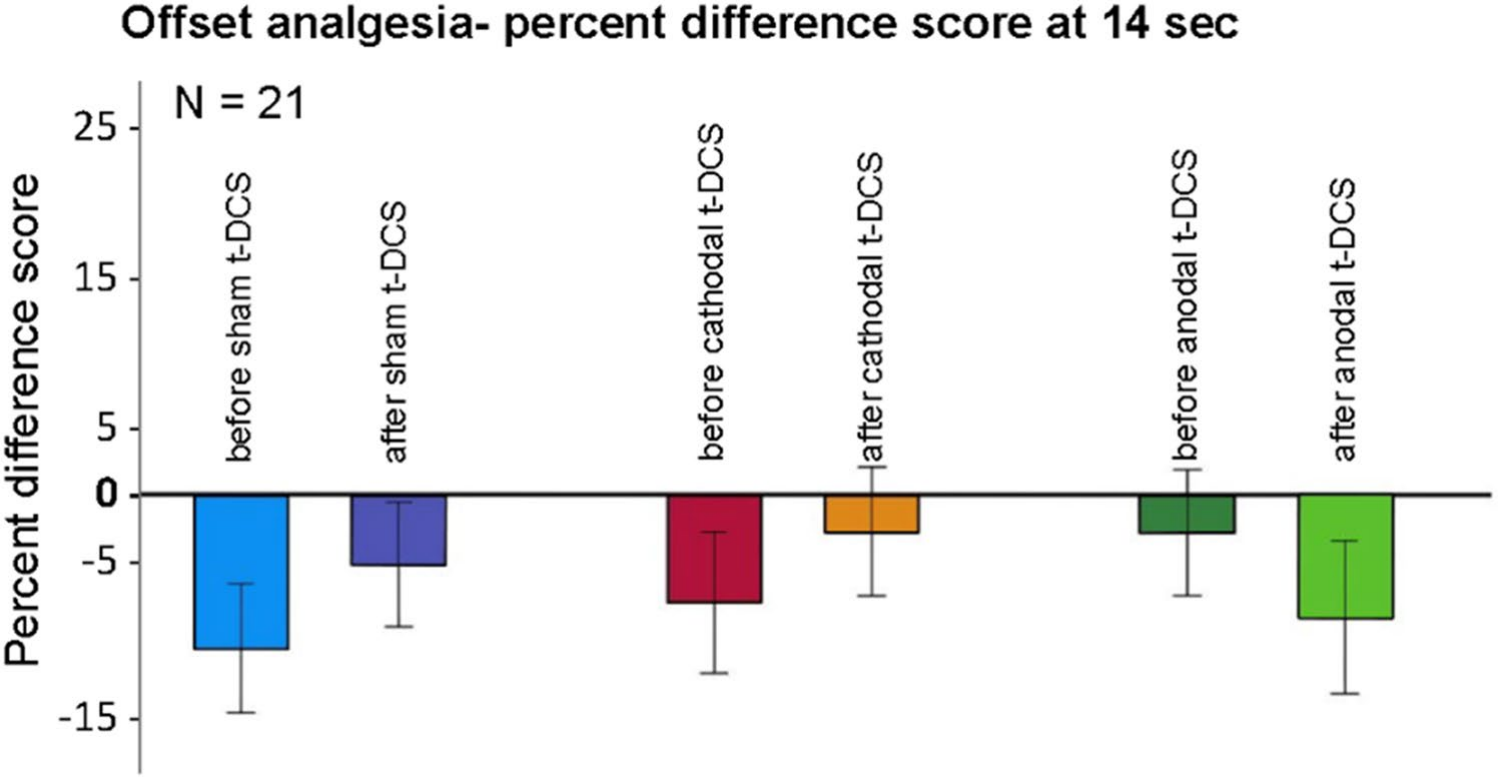
t-DCS effects on offset analgesia. Offset analgesia was quantified as percent difference scores at 14 s (see “Methods”). There was a trend for an interaction between time and t-DCS type (*P* = 0.085), due to a significantly increased offset analgesia after anodal compared to sham t-DCS (*P* = 0.023)

Results are shown in Supp. Table 2 and Fig. 3. ANOVA revealed a trend for an interaction between time and t-DCS type for the offset analgesia effect (*F* [2, 40] = 2.619; *P* = 0.085). Exploratory subordinate ANOVAs revealed a significantly increased offset analgesia after anodal (5.7% ± 15.9%) compared to sham (− 5.6% ± 26.0%) t-DCS (*F* [1, 20] = 6.058; *P* = 0.023) (Fig. 3).

### Conditioned Pain Modulation

The CPM effect was significant, amounting to an average reduction in pain rating by 8.6% ± 9.7% (*F* [1, 20] = 35.381; *P* < 0.001); however, it did not significantly differ between t-DCS types (*F* [2, 40]= 0.174; *P* = 0.841, Supp. Table 3).

### Blinding Check

After anodal, cathodal, and sham t-DCS, 11, 15, and 11 of the 21 participants indicated to believe to have received true t-DCS, respectively. There were no significant differences between t-DCS types (χ^2^(2) = 1.8; *P* = 0.369).

### Adverse Effects

Apart from itching under the electrodes and one instance of moderate headache on the day following stimulation in a participant with pre-existing episodic migraine, no adverse effects of t-DCS were reported.

## Discussion

Results of the present study suggest that cathodal (inhibitory) t-DCS increased pain perception and reduced endogenous pain inhibition while anodal (excitatory) t-DCS increased endogenous pain inhibition. These results are principally in line with the idea of an activation of endogenous pain inhibition by cerebellar activation [2, 15, 16]. However, effects were small and evident only at an exploratory analysis level.

### Cerebellar t-DCS Effects on Pain Perception

Cathodal t-DCS has the potential to reduce cortical excitability [31]. In our experiments, cathodal cerebellar t-DCS seemed to increase pain perception, as shown by a trend for a decrease in electrical pain thresholds. However, this was evident only immediately after t-DCS, not at 30 or 60 min. Also, electrical and heat pain ratings were not affected. In summary, effects regarding pain perception were small and need to be regarded with caution. In a previous study, cathodal cerebellar t-DCS significantly increased VAS scores in response to laser stimulation at the hand up to 60 min after t-DCS, while anodal t-DCS did the opposite [11]. Another study, with the stimulating electrode placed over the right cerebellar hemisphere and the reference over the buccinator muscle, found increased electrical pain thresholds after anodal cerebellar t-DCS, but no effect of cathodal t-DCS [12]. Together, our and the previous results point in the same direction, suggesting that inhibition of cerebellar activity increases pain perception while excitation does the opposite. This also fits with our previous result that chronic cerebellar infarction patients show increased pain perception [2], and with the findings of a clinical study where 5 sessions of anodal cerebellar t-DCS reduced paroxysmal pain (but not phantom limb or stump pain) in phantom limb pain patients [24].

### T-DCS Effects on Supraspinal Nociception as Quantified by Late SEPs

We found increased N120 amplitudes after cathodal stimulation. As the N120 component of late SEPs represents parietal operculum and insula activity, this fits with an increased supraspinal nociception [32]. Again, it must be noted that the result was evident only in exploratory analysis and only for the time point 30 min after stimulation. Previous studies using laser evoked potentials (LEPs) showed cathodal cerebellar t-DCS to increase N1 and N2/P2 amplitudes, while anodal t-DCS had the opposite effect [11, 13]. Since the LEP N1 component likely corresponds to the SEP N120 [33, 34], these results are partially reproduced by our findings. Together, our and the previous results again point in the same direction, suggesting that inhibition of cerebellar activity by cathodal t-DCS increases supraspinal nociception. This is also consistent with increased pain perception as described above.

### T-DCS Effects on Endogenous Pain Modulation

This is the first study assessing the effect of cerebellar t-DCS on measures of endogenous pain modulation, namely, the RIII, OA, and CPM. We found an increase in RIII reflex area immediately after cathodal t-DCS and an increase in offset analgesia after anodal t-DCS, but no effect on CPM. These results again must be viewed with caution because they were evident only in the exploratory analysis.

The RIII reflex is a spinally mediated nociceptive reflex modulated by descending pain inhibitory pathways [35, 36]. An increase in RIII size after cathodal t-DCS therefore suggests a reduction in descending pain inhibition, especially when found in parallel with an increase in pain perception as suggested by the above results. Offset analgesia (OA) is a disproportionate decrease in pain perception after a small reduction in nociceptive stimulation, and it is thought to represent another facet of endogenous pain inhibition, possibly also involving descending pathways [37, 38]. In addition, cerebellar activity has been demonstrated during OA [38], potentially making OA especially adequate to detect effects of cerebellar t-DCS. An increase in OA after anodal (excitatory) t-DCS as seen here is compatible with an increase in pain inhibitory mechanisms. This is also consistent with our previous study showing reduced OA in chronic cerebellar infarction patients [2].

Previous animal studies have shown that electrical or chemical stimulation of the cerebellum alters responses of spinal nociceptive neurons [15, 16], also consistent with the notion that the cerebellum can affect endogenous/descending pain modulatory pathways.

In summary, two of three measures of endogenous pain modulation suggested that cathodal cerebellar t-DCS reduces while anodal t-DCS increases endogenous (possibly descending) pain inhibition. Together, the present and previous results would be consistent with the hypothesis that cerebellar activation reduces pain perception via probably an effect on endogenous/descending pain inhibitory pathways while cerebellar inhibition does the opposite. This would be an expansion of the notion that reduction of pain perception by anodal (excitatory) cerebellar t-DCS is due to strengthening of the tonic inhibitory cerebellar influence over cortical areas (“cerebellar brain inhibition”) [14].

### Strengths and Limitations

The greatest strength of the study is the elaborate sham-controlled, double-blind cross-over design using 4 time points and several measures of pain perception. However, this also makes multiple testing a larger problem, especially when effects are small. Indeed, our effects were significant only at an exploratory level. The sample size was principally adequate, even somewhat larger than that of previous studies that included 14 to 16 participants [11, 12].

There are several possible reasons why we found only small effects. The occipital bone over the cerebellum is very thick [39] which decreases t-DCS effects [40]. In addition, modeling studies show that t-DCS preferentially affects dorsal cerebellar areas [7, 41], while areas involved in pain perception are mostly located ventrally [5]. We positioned electrodes (reference over the upper arm) according to the previous publication showing largest t-DCS effects [11]. However, using a buccinator reference may improve access of ventral cerebellar areas [42, 43]. Maybe new methods such as high-intensity t-DCS can in the future improve access of ventral cerebellar areas [14]. More focal stimulation techniques may also allow to more specifically dissect pathways underlying cerebellar modulation of pain. Another limitation of our study is that we did not directly assess activation of cerebellar brain inhibition by cerebellar t-DCS, e.g., by measuring motor-evoked potentials [44].

In addition, individual differences may affect susceptibility to cerebellar t-DCS modulation of pain. For example, the effect of cerebellar t-DCS on pain perception and LEPs was blunted in subjects with high hypnotizability [13]. We did not test hypnotizability levels in our sample.

Moreover, a single t-DCS session as used here and in previous experimental studies might be insufficient to induce a stable effect. Clinical studies showing t-DCS effects on pain perception use at least 5 sessions and often many more [14]. On way to deal with that could be the amelioration of the effects of t-DCS by concomitant use of drugs [45]. Also, it has been a general experience that t-DCS effects are more difficult to observe in healthy participants compared to chronic pain patients [46]. It is a matter of discussion if pain processing is altered in chronic pain patients in a way making it more susceptible to modulation by t-DCS.

In addition, it must be mentioned that the measures of endogenous pain modulation and supraspinal nociception used here only reflect facets of these phenomena and are subject to confounding. This may be especially evident for the electrophysiological measures. Both the RIII reflex and the N120 are not purely nociceptive, and the RIII reflex arch contains spinal interneurons and motor neurons [35, 36, 47]. Dissociation of effects on pain perception and RIII reflexes suggest that not every change in RIII size reflects a change in descending pain inhibition [48, 49]. In the present study, we used SEPs for assessment of supraspinal nociception, because they can be assessed together with the RIII reflex. However, it must be acknowledged that LEPs would have allowed a more nociceptive-specific assessment of supraspinal nociception.

### Conclusion

Our results suggest that cathodal (inhibitory) cerebellar t-DCS increased pain perception and reduced endogenous pain inhibition while anodal (excitatory) t-DCS did the opposite. This is compatible with previous results on cerebellar influences on pain perception, and tentatively supports our hypothesis that the cerebellum may be involved in pain modulation via endogenous, including descending pain pathways. Although effects were small and unlikely to be clinically significant, results nonetheless contribute to the increasing understanding of the interactions between the cerebellum and pain perception, and encourage future studies, preferably using improved methods of cerebellar stimulation.

### Supplementary Information

Below is the link to the electronic supplementary material.

Supplementary file1 (PDF 244 KB)

## Abbreviations

AUC: Area under the curve
BDI: Beck Depression Inventory
CPM: Conditioned pain modulation paradigm
DC: Direct current
LEP: Laser evoked potential
NRS: Numerical rating scale
OA: Offset analgesia
PAG: Periaqueductal gray
PCS: Pain Catastrophizing Scale
PSQ: Pain Sensitivity Questionnaire
RIII reflex: Polysynaptic spinal nociceptive flexor reflex
RR: Ruth Ruscheweyh, supervisor
RS: Regina Stacheneder, investigator
SEPs: Late somatosensory evoked potentials
STAI: State-Trait Anxiety Inventory
t-DCS: Transcranial direct current stimulation
ct-DCS: Cerebellar transcranial direct current stimulation
VAS: Visual analog scale
