# Supplementary material for: Effects of Transcranial Direct Current Stimulation (t-DCS) of the Cerebellum on Pain Perception and Endogenous Pain Modulation: a Randomized, Monocentric, Double-Blind, Sham-Controlled Crossover Study
Source: Cerebellum. 2022 Dec 8;22(6):1234–42. doi: 10.1007/s12311-022-01498-x (PMC10657278; doi:10.1007/s12311-022-01498-x)
Supplement: Supplementary file 1 — Supplementary file1 (PDF 244 KB) [file 12311_2022_1498_MOESM1_ESM.pdf]

Supplementary Figure 1

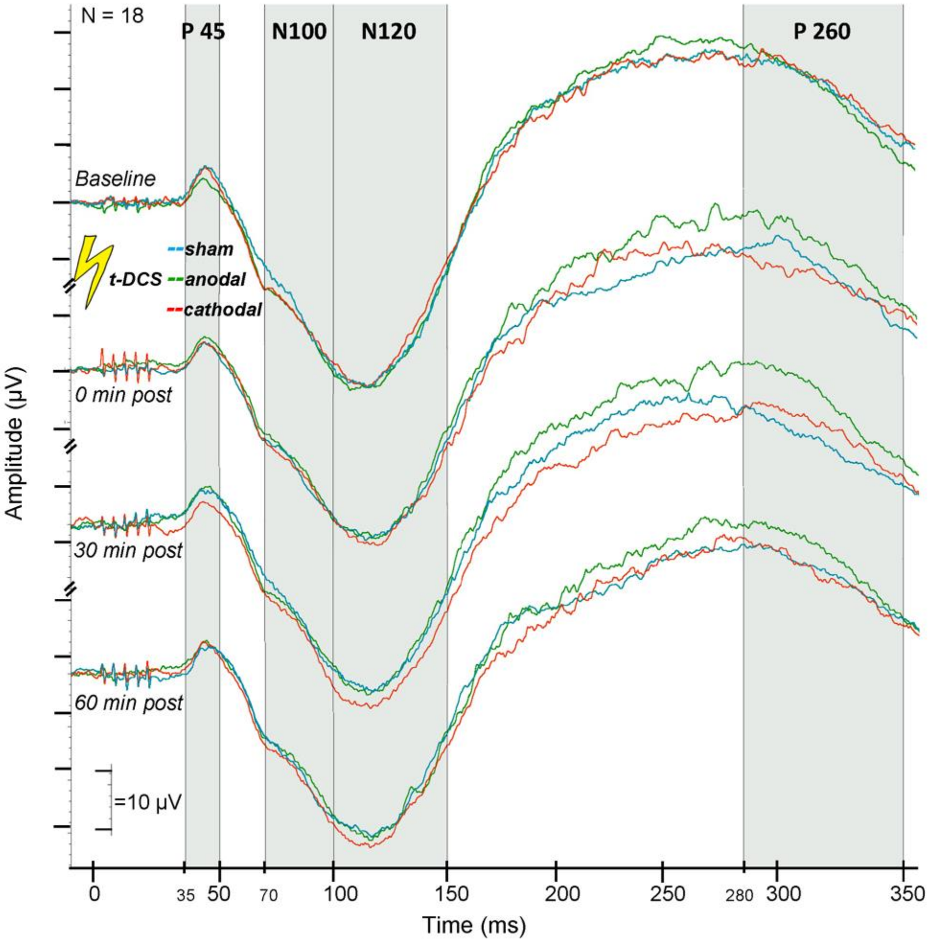

Supplementary Figure 1. t-DCS effect on SEPs. Mean SEP curves over all participants are shown (n = 21). Analysis windows for the four components (P45, N100, N120, P260) are illustrated. Exploratory analysis showed a significant increase of the N120 amplitude after cathodal compared to anodal stimulation at 30 min compared to baseline (P= 0.007).

Supplementary Table 1

	anodal t-DCS		***cathodal t-DCS***		***sham t-DCS***	
N = 21	pre t-DCS	post t-DCS	pre t-DCS	post t-DCS	pre sham	post sham
44°C	1.2 ± 1.2	1.4 ± 1.4	1.7 ± 1.6	1.4 ± 1.5	1.3 ± 1.4	1.3 ± 1.4
45°C	2.1 ± 1.4	1.9 ± 1.8	2.3 ± 1.7	1.9 ± 1.5	1.9 ± 1.5	1.7 ± 1.6
46°C	3.2 ± 1.7	3.3 ± 2.2	3.9 ± 1.9	3.5 ± 1.9	3.6 ± 1.7	3.5 ± 1.8
47°C	5.3 ± 1.8	5.7 ± 2.0	5.6 ± 1.9	5.5 ± 1.9	5.3 ± 1.9	5.3 ± 2.1
48°C	6.8 ± 2.0	6.9 ± 2.3	7.1 ± 1.9	6.8 ± 1.9	6.6 ± 2.0	6.7 ± 2.2

Supplementary Table 1. Heat pain intensity ratings (NRS). Values are mean ± standard deviation.

Supplementary Table 2

	anodal t-DCS		***cathodal t-DCS***		***sham t-DCS***	
N = 21	pre t-DCS	post t-DCS	pre t-DCS	post t-DCS	pre sham	post sham
4 sec (ref)	0 ± 0	0 ± 0	0 ± 0	0 ± 0	0 ± 0	0 ± 0
9 sec	39.6 ± 26.2	36.1 ± 33.4	39.3 ± 25.9	42.2 ± 34.5	37.6 ± 28.0	42.2 ± 28.7
14 sec	**-2.5 ± 19.4**	**-8.2 ± 23.6**	**-7.3 ± 21.7**	**-2.5 ± 19.7**	**-10.3 ± 19.8**	**-4.7 ± 19.1**
19 sec	2.7 ± 17.5	-6.3 ± 17.6	-0.5 ± 20.6	-1.9 ± 15.2	-4.0 ± 19.5	-2.5 ± 16.0
24 sec	5.0 ± 13.0	-4.7 ± 14.8	6.4 ± 16.6	-1.8 ± 12.4	1.4 ± 15.0	-0.7 ± 15.7
29 sec	7.8 ± 11.4	-3.9 ± 13.8	6.1 ± 16.4	1.0 ± 10.6	5.1 ± 14.0	4.9 ± 20.9

Supplementary Table 2. Offset analgesia. Effects of t-DCS (anodal/cathodal/sham) on percentage difference scores at each time point (4, 9, 14, 19, 24, 29 sec) of the 30s heat stimulus. Percent difference scores are obtained by normalizing to the 4 sec rating and calculating the difference between offset and control runs (offset minus control). The offset analgesia effect was defined as the percent difference score at 14 sec, marked in bold.

Supplementary Table 3

N = 21	***post anodal t-DCS***	***post cathodal t-DCS***	***post sham t-DCS***
NRS before conditioning	4.50 ± 1.65	4.47 ± 1.76	4.45 ± 1.67
NRS during conditioning	3.56 ± 1.81	3.68 ± 1.78	3.61 ± 1.99
CPM effect	**-0.95 ± 1.15**	**-0.79 ± 0.87**	**-0.84 ± 0.90**

Supplementary Table 3. CPM effect (measured after t-DCS). Test stimulus pain intensity ratings (NRS) before and during conditioning stimulus are given. The CPM effect was calculated as (NRS during – NRS before).
